# Genomic landscape of advanced prostate cancer patients with *BRCA1* versus *BRCA2* mutations as detected by comprehensive genomic profiling of cell-free DNA

**DOI:** 10.3389/fonc.2022.966534

**Published:** 2022-09-15

**Authors:** Umang Swami, Raquel Mae Zimmerman, Roberto H. Nussenzveig, Edgar Javier Hernandez, Yeonjung Jo, Nicolas Sayegh, Sergiusz Wesolowski, Lesli A. Kiedrowski, Pedro C. Barata, Gordon Howard Lemmon, Mehmet A. Bilen, Elisabeth I. Heath, Lakshminarayan Nandagopal, Hani M. Babiker, Sumanta K. Pal, Michael Lilly, Benjamin L. Maughan, Benjamin Haaland, Mark Yandell, Oliver Sartor, Neeraj Agarwal

**Affiliations:** ^1^ Division of Oncology, Department of Internal Medicine, Huntsman Cancer Institute, University of Utah, Salt Lake City, UT, United States; ^2^ Human Genetics, University of Utah, Salt Lake City, UT, United States; ^3^ Division of Oncology and Department of Population Health Sciences, Huntsman Cancer Institute, University of Utah, Salt Lake City, UT, United States; ^4^ Department of Medical Affairs, Guardant Health, Redwood City, CA, United States; ^5^ Deming Department of Medicine, Section of Hematology/Oncology, Tulane University Medical School, New Orleans, LA, United States; ^6^ Department of Hematology and Medical Oncology, Winship Cancer Institute of Emory University, Atlanta, GA, United States; ^7^ Department of Oncology, Barbara Ann Karmanos Cancer Institute, Wayne State University School of Medicine, Detroit, MI, United States; ^8^ Department of Medical Oncology, University of Alabama Medical Center, Birmingham, AL, United States; ^9^ Department of Medical Oncology, Mayo Clinic Florida, Jacksonville, FL, United States; ^10^ Genitourinary Oncology, City of Hope Comprehensive Cancer Center, Duarte, CA, United States; ^11^ Division of Hematology and Oncology, Department of Medicine, Medical University of South Carolina, Charleston, SC, United States; ^12^ Tulane Cancer Center, Tulane Medical School, New Orleans, LA, United States

**Keywords:** *BRCA1 vs. BRCA2 landscape by cfDNA BRCA1*, *BRCA2*, ctDNA, advanced prostate cancer, machine learning

## Abstract

*BRCA1*-mutated prostate cancer has been shown to be less responsive to poly (ADP-ribose) polymerase (PARP) inhibitors as compared to *BRCA2*-mutated prostate cancer. The reason for this differential response is not clear. We hypothesized this differential sensitivity to PARP inhibitors may be explained by distinct genomic landscapes of *BRCA1* versus *BRCA2* co-segregating genes. In a large dataset of 7,707 men with advanced prostate cancer undergoing comprehensive genomic profiling (CGP) of cell-free DNA (cfDNA), 614 men harbored *BRCA1* and/or *BRCA2* alterations. Differences in the genomic landscape of co-segregating genes was investigated by Fisher’s exact test and probabilistic graphical models (PGMs). Results demonstrated that *BRCA1* was significantly associated with six other genes, while *BRCA2* was not significantly associated with any gene. These findings suggest *BRCA2* may be the main driver mutation, while *BRCA1* mutations tend to co-segregate with mutations in other molecular pathways contributing to prostate cancer progression. These hypothesis-generating data may explain the differential response to PARP inhibition and guide towards the development of combinatorial drug regimens in those with *BRCA1* mutation.

## Introduction

Poly (ADP-ribose) polymerase (PARP) inhibitors such as olaparib and rucaparib are currently approved for patients with metastatic castration-resistant prostate cancer (mCRPC) with *BRCA1* and *BRCA2* alterations (1, 2). However, multiple studies have noted that patients with prostate cancer harboring *BRCA2* mutations are more responsive to PARP inhibitors compared to *BRCA1* mutation-positive patients. In a pooled analysis of 5 studies, men with *BRCA1* mutated prostate cancer compared to *BRCA2* mutated had a lower PSA_50_ response rate (23.8% vs. 65.2%), lower overall response rate (26.3% vs. 50%) and a lower median radiographic progression-free survival (4.1 months vs. 10.1 months) (3). The reason for this differential efficacy is not clear. We hypothesized this differential efficacy may be explained by distinct genomic landscapes of prostate cancer harboring *BRCA1* versus *BRCA2* mutation.

## Materials and methods

All patients with advanced prostate cancer who underwent comprehensive genomic profiling (CGP) of cell-free DNA (cfDNA) by a Clinical Laboratory Improvement Amendments (CLIA)-certified, College of American Pathologists (CAP)-accredited laboratory (Guardant360, Redwood City, CA) between 11/2016 to 8/2020 were eligible. First available cfDNA CGP results from consecutive patients with advanced prostate cancer tested were evaluated for the presence of *BRCA1/2* mutations. This included all cfDNA somatic alterations defined as reportable by clinical testing parameters. All variants of unknown significance were excluded from the analysis. Frameshift and nonsense mutations were included as pathogenic. **Supplementary Figure 1** provides a graphical representation of the cohort selection process and number of patients excluded at each step.

The prevalence of *BRCA1* and *BRCA2* mutations in our cohort of patients was compared to published reports by the chi-squared test. Pairwise associations of mutation-positive *BRCA1* or *BRCA2* genes with other mutated genes was independently assessed by Fisher’s exact test, and p-values were adjusted for false discovery rate* (FDR) to control multiple testing. Statistical significance was defined as a p-value ≤ 0.05.

Multilevel gene interdependencies between *BRCA1* or *BRCA2* were assessed using a combination of two probabilistic graphical model (PGM) machine learning approaches. To account for the high computational cost of the PGM dependence structure discovery, we identified the nearest *BRCA1* or *BRCA2* neighboring genes by an approximate PGM structure finding algorithm (4).

Once the candidate genes were identified, we used the “exact” DP-A* (5) structure search algorithm provided by the bnlearn R package (4). For the parameter learning and inference we used the default loopy belief propagation algorithm. Visualizations were done using LaTeX (https://texdoc.org/serve/pgfmanual.pdf/0). All significant relations were captured by the PGM.

## Results

**Figure 1:**
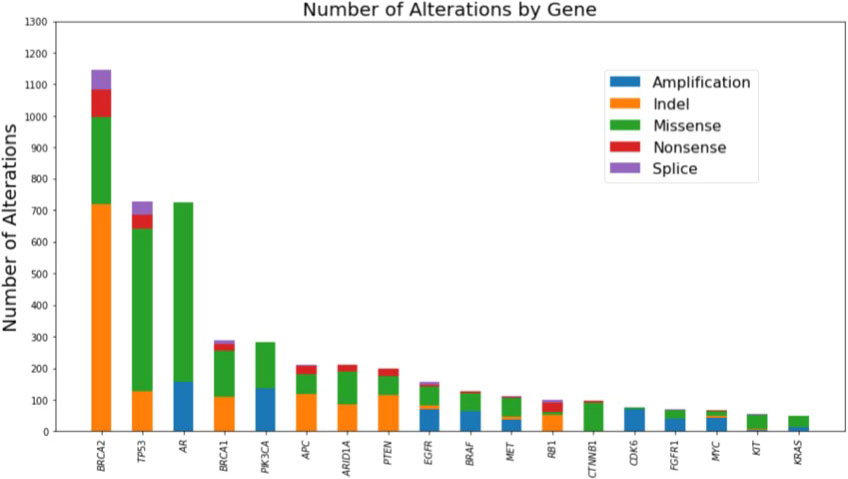
Mutational landscape of genes with alterations present in >=5% of the cohort as detected by comprehensive genomic profiling of cell-free DNA of 614 unique patients with advanced prostate cancer harboring *BRCA1* or *BRCA2* mutations.

CGP of cfDNA from 7,707 unique men with advanced prostate cancer was available to assess the presence of mutations in *BRCA1* and/or *BRCA2*. The median age for the total cohort was 72 years (interquartile range 65-78 years). Pathogenic mutations in *BRCA1/2* were found in 614 of 7,707 unique patients. The frequency of alterations in *BRCA1* (4.6%) and *BRCA2* (7.97%) detected in cfDNA was similar to what has been reported from CGP of primary tissue (**Supplementary Table 1**) (6, 7). The cfDNA mutational landscape of genes with alterations present in ≥5% of these 614 unique patients with advanced prostate cancer is presented in **Figure 1**.

A significant association between mutation-positive *BRCA1* and alterations in 6 other genes (*ERBB2*, *NOTCH1*, *AKT1*, *MTOR*, *ARID1A*, and *EGFR*) was identified by Fisher’s exact test (**Table 1**). In contrast, there were no significant associations between mutation-positive *BRCA2* and any other altered gene.

**Table 1:** Statistical analysis of co-segregation of genes with *BRCA1* or *BRCA2*.

Gene	*BRCA1+* p-value*	*BRCA2+* p-value*
*ERBB2*	0.001	NS
*NOTCH 1*	0.002	NS
*AKT1*	0.011	NS
*MTOR*	0.014	NS
*ARID 1A*	0.008	NS
*EGFR*	0.048	NS

NS, not significant; *Fisher’s exact p-values adjusted for false discovery rate.

**Figure 2:**
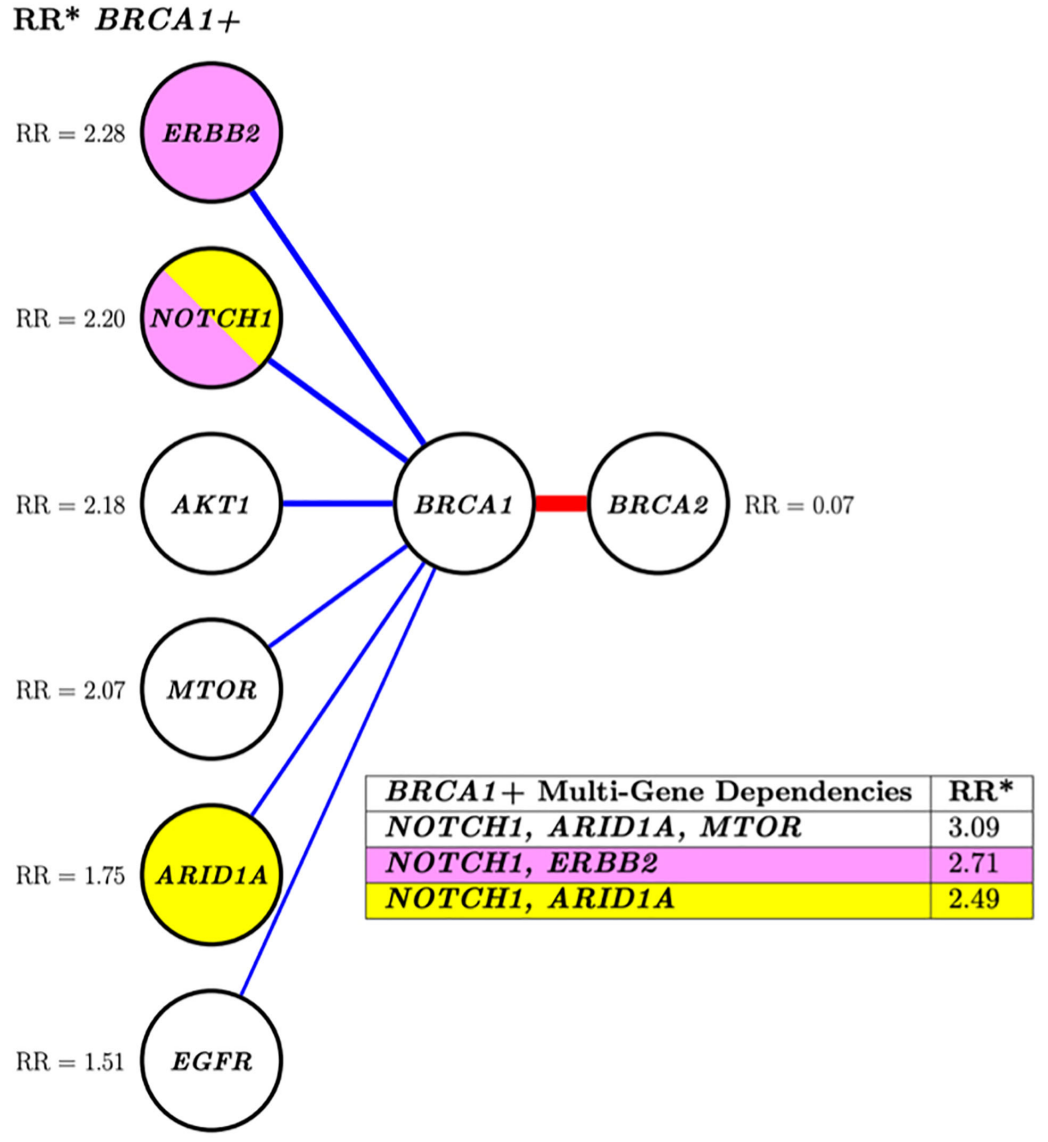
Conditional risk landscape visualization. There is an increased association of *BRCA1* (left) versus *BRCA2* (right) with somatically mutated genes with pathogenic variants. The table inset displays the risk of having mutations in the genes lkisted in the table if the patient also has a *BRCA1* muted gene. *RR, Related risk of co-segregation of gene of interest and *BRCA1* and/or *BRCA2* compared to *BRCA1* and/or *BRCA2* in the absence of the gene. Each node represents a variable, and each edge (line) indicates a dependence between variables. Blue indicates increased risk of the outcome of co-segregation and red indicates decreased risk of the outcome of co-segregation. The width of the colored lines is scaled by strength of association. The pairs of yellow and pink shaded nodes correspond to the yellow and pink rows of the table (lower right) which states the associated relative risk of the pair in relation to the outcome.

Fifteen altered genes were identified as nearest neighbors to mutation-positive *BRCA1/2* by an approximate PGM structure finding algorithm (data not shown) and selected for further analysis by the costly, “exact” DP-A* (5) structure search algorithm. The PGM network analysis demonstrated positive interdependencies between pathogenic variants in *BRCA1* and 6 other altered genes. A negative association between mutation-positive *BRCA1* and *BRCA2* was identified as indicated by a relative risk for co-segregation of 0.07 (**Figure 2**).

Investigation of more complex multi-variant interactions, such as those between *BRCA1* and the various mutated gene combinations of *NOTCH1, ARID1A*, and *ERBB2* (**Figure 2**, table inset) revealed increased relative risks for these multi-level associations. For instance, the relative risk of patients with a *BRCA1* mutation and a *NOTCH1* alteration was 2.20, this risk increased to 3.09 if *ARID1A* and *MTOR* were also mutated. This type of multi-level dependency cannot be investigated by conventional pairwise statistics or regression analysis. Overall, both analyses showed a greater likelihood of multiple gene segregation with *BRCA1* versus *BRCA2*.

## Discussion

Our results demonstrate that *BRCA1* mutation in patients with advanced prostate cancer was significantly associated with 6 other genes, while *BRCA2* was not significantly associated with any gene. The higher number of significant concurrently mutated genes in other molecular pathways may explain the decreased efficacy of PARP inhibitors in prostate cancer harboring *BRCA1* mutations compared to those with *BRCA2* mutation. For example, *ERBB2* alterations co-segregate strongly with *BRCA1* and are known to be associated with tumor aggressiveness in primary prostate cancer, tumor progression and invasion in advanced prostate cancer, and shorter time to castration-resistance (8). Similarly, alterations in *NOTCH1* also co-segregate with *BRCA1*. Dysregulated activation of *NOTCH1* promotes development of prostate cancer metastasis and castration-resistance by altering signaling through multiple oncogenic pathways including AKT, Myc, and Ras/Raf/MAPK pathway (9). Using both Fisher’s exact test and PGMs we demonstrate that the presence of mutated genes, such as *ERBB2* and *NOTCH1*, are more likely to co-occur in patients harboring *BRCA1* mutations. Furthermore, the use of PGMs, which capture multivariate, multi-level dependencies, revealed increased relative risks with the combination of two or more pathogenically mutated genes in *BRCA1* mutation-positive patients.

Our results show a higher prevalence of *BRCA1* alterations and a slightly lower prevalence of *BRCA2* alterations than previously reported, as described in **Supplementary Table 1**. These differences may be due to three reasons. First, prior studies which have sequenced tumor tissue are mostly from primary prostate and less frequently from metastatic sites. Liquid biopsies in patients with prostate cancer are usually done in clinics once multiple therapies such as androgen-receptor axis targeting agents and taxanes have been utilized. In this situation, tissue biopsies are generally difficult due to bone predominant disease and there are not many studies to elucidate the tumor mutation profile in this scenario. Therefore, it is likely that patients with advanced disease after progression on standard therapies are enriched with BRCA1 mutations. Second, liquid biopsies combine the mutation profile of tumors across all metastatic sites. Again, it is currently unknown how the metastatic sites differ from primary in terms of mutations, and our results may be a reflection of it. Third, as acknowledged below in limitations we are unable to determine if the origin of mutations in cfDNA in our study is from tumor or germline and this may also have increased the incidence of *BRCA1* alterations as compared to historical tissue somatic testing results.

Despite recent advances and the approval of multiple agents for the treatment of advanced prostate cancer, the disease remains lethal (10, 11). After disease progression on an androgen receptor targeted therapy, the median overall survival of these patients is only two years (12). Therefore, it is critical to discover molecular pathways of disease progression and develop novel drugs targeting these pathways to improve outcomes. One way to identify these pathways is to obtain tumor biopsies upon disease progression on a given therapy. However, in most patients with metastatic prostate cancer, bones remain the only site of metastatic disease, making these tumor biopsies impractical in the real-world setting. In many others, a metastatic site biopsy is considered unsafe, expensive, invasive, and not desirable. In this context, interrogation of cfDNA to identify these molecular pathways of disease resistance is an attractive alternative.

We and others have previously shown the feasibility of utilizing CGP of the cfDNA in patients with advanced prostate cancer (13, 14) to identify disease resistance pathways. Although two PARP inhibitors (olaparib and rucaparib) were only recently approved for mCRPC treatment, this field is expected to undergo a rapid evolution with more PARP inhibitors either as single agents or in novel combinations predicted to be approved shortly. Against this backdrop, it has become critical to identify mechanisms of differential response and disease resistance to PARP inhibitors concerning various underlying homologous recombination repair mutations. The current study attempts to elucidate the molecular mechanism of differential response to PARP inhibitors in men with advanced prostate cancer harboring *BRCA1* versus *BRCA2* mutation. Our results, upon external validation in independent cohorts with available cfDNA and tumor tissue DNA, may guide the development of novel treatment regimens for these patients.

The limitations of this study include the lack of clinical annotation such as the disease state and treatment exposure (including potential responses to PARP inhibitor therapy), as well as the inability to definitively determine the origin of mutations identified in cfDNA (e.g. tumor versus germline versus hematopoietic). Strengths of the study include the number of patients and centers included and the dataset’s real-world nature. These hypothesis-generating data reveal differential genomic signatures associated with *BRCA1* as compared to *BRCA2* which may translate in the development of novel combinatorial regimens for patients in the future.

## Data availability statement

The datasets generated and/or analyzed for the current study are not publicly available, as they are derived from commercial testing. This data may be made available under a fully executed data use agreement. Requests to access these datasets should be directed to Lesli Kiedrowski, lkiedrowski@guardanthealth.com.

## Ethics statement

The studies involving human participants were reviewed and approved by University of Utah IRB. Written informed consent for participation was not required for this study in accordance with the national legislation and the institutional requirements.

## Author contributions

Conception and design: US, RN, NA. Acquisition of data: LK, RN. Analysis and interpretation of data: US, RZ, RN, EJH, BH, MY, NA. Drafting of the manuscript: US, RZ, RN, NA. Critical revision of the manuscript for important intellectual content: US, RZ, RN, EJH, YJ, NS, SW, LK, PB, GL, MB, EIH, LN, HB, SP, ML, BM, BH, MY, OS, NA. Statistical analysis: US, RZ, RN, EJH, YJ, BH. All authors contributed to the article and approved the submitted version.

## Conflict of interest

NA reports consultancy to Astellas, Astra Zeneca, Aveo, Bayer, Bristol Myers Squibb, Calithera, Clovis, Eisai, Eli Lilly, EMD Serono, Exelixis, Foundation Medicine, Genentech, Gilead, Janssen, Merck, MEI Pharma, Nektar, Novartis, Pfizer, Pharmacyclics, and Seattle Genetics; and additionally reports institutional research funding from Astra Zeneca, Bavarian Nordic, Bayer, Bristol Myers Squibb, Calithera, Celldex, Clovis, Eisai, Eli Lilly, EMD Serono, Exelixis, Genentech, Glaxo Smith Kline, Immunomedics, Janssen, Medivation, Merck, Nektar, New Link Genetics, Novartis, Pfizer, Prometheus, Rexahn, Roche, Sanofi, Seattle Genetics, Takeda, and Tracon. LK is an employee and stockholder of Guardant Health. PB declares grants or contracts from Merck, Seagen, Blue Earth Diagnostics, Pfizer, and EMD Serono; consulting fees from Dendreon, Pfizer, Caris Life Sciences, Astellas, Eisai, Janssen, EMD Serono, Seattle Genetics, Bristol-Myers Squibb, Bayer, and Guardant Health; payments or honoraria from Caris Life Sciences, Bayer, and Pfizer; and participation on boards for Bristol-Myers Squibb, Seagen, Astellas, Eisai, Janssen, EMD Serono, Dendreon, Pfizer, Seattle Genetics, Bayer, and Guardant Health. MB declares consulting fees from Exelixis, Bayer, Bristol-Myers Squibb, Eisai, Pfizer, AstraZeneca, Janssen, Calithera Biosciences, Genomic Health, Nektar, EMD Serono, Seagen, and Sanofi and institutional research support from Merck, Xencor, Bayer, Bristol-Myers Squibb, Genentech/Roche, Seagen, Incyte, Nektar, AstraZeneca, Tricon Pharmaceuticals, Genome & Company, AAA, Peloton Therapeutics, and Pfizer for work performed outside the current study. EIH has received honoraria from Bayer, Sanofi, and Seattle Genetics; acted as a consultant/advisor for Astellas Pharma; is an Advisory Board and/or Speakers’ Bureau member for AstraZeneca, Bayer, Bristol-Myers Squibb, Sanofi; and has received paid travel from Astellas Pharma, Caris Life Sciences, Sanofi, and Seattle Genetics; in addition, her institution has received research funding from Astellas Pharma, AstraZeneca, Boehringer Ingelheim, Bristol-Myers Squibb, Caris Life Sciences, Celgene, Celldex, Corcept Therapeutics, Curementa, Dendreon, eFFECTOR Therapeutics, Esanik, Fortis Therapeutics, Genetech/Roche, GlaxoSmithKline, Ignyta, Inovio Pharmaceuticals, Medivation, Merck Sharp & Dohme, Merck, Millennium, Oncolys BioPharma, Plexxicon, Seattle Genetics, Synta, Tokai Pharmaceuticals, and Zenith Epigenetics. HB Consulting or Advisory Role: Endocyte, Celgene, Idera, Myovant Sciences Speakers' Bureau: Guardant Health. SP reports personal fees from F. Hoffman-La Roche outside the submitted work, as well as research funding to his institution from Eisai, Genentech, Roche, Exelixis, and Pfizer; and reports a consulting/advisory role for Novartis, Medivation, Astellas Pharma, Pfizer, Aveo, Myriad, Genentech, Exelixis, and Bristol-Myers Squibb. US reports consultancy to Astellas, Exelixis and Seattle Genetics and research funding to institute from Janssen, Exelixis and Astellas/Seattle Genetics. OS is a consultant for Advanced Accelerator Applications, Astellas, AstraZeneca, Bayer Blue Earth Diagnostics Inc., Bavarian, Nordic, Bristol, Myers, Squibb, Clarity, Pharmaceuticals, Clovis, Constellation, Dendreon, EMD, Serono, Fusion, Janssen, Myovant, Myriad, Noria, Therapeutics, Inc., Novartis, Noxopharm, Progenics, POINT, Biopharma, Pfizer, Sanofi, Tenebio, Telix, Theragnostics, Dendreon, Endocyte, Innocrin, Invitae, Merck, and SOTIO; research funding from Advanced Accelerator Applications, AstraZeneca, Bayer, Invitae, and Merck. BH reports receiving travel assistance from Flatiron Health, and served as a consultant for AstraZeneca, Value Analytics, National Kidney Foundation, and Prometic Life Sciences. MY is a stock holder or has received stock option awards from Fabric Genomics Inc. and has received consulting fees from Fabric Genomics Inc. BM has consulted for Janssen Oncology, Exelixis, Tempus, Peloton Therapeutics and Astellas. RN has consulted for Tempus.

The remaining authors declare that the research was conducted in the absence of any commercial or financial relationships that could be construed as a potential conflict of interest.

## Publisher’s note

All claims expressed in this article are solely those of the authors and do not necessarily represent those of their affiliated organizations, or those of the publisher, the editors and the reviewers. Any product that may be evaluated in this article, or claim that may be made by its manufacturer, is not guaranteed or endorsed by the publisher.
