# Supplementary material for: Genomic landscape of advanced prostate cancer patients with BRCA1 versus BRCA2 mutations as detected by comprehensive genomic profiling of cell-free DNA
Source: Front Oncol. 2022 Sep 15;12:966534. doi: 10.3389/fonc.2022.966534 (PMC9521349; doi:10.3389/fonc.2022.966534)

**Supplementary Table 1:** Comparison of comprehensive genomic profiling of cell-free DNA from Guardant dataset with comprehensive genomic profiling of primary and metastatic tissue from historical datasets.

| **Gene** | **Study** | **Sample** | **Prevalence (%)** | **95% Confidence Interval** | **Pt num** | **p-value** |
| --- | --- | --- | --- | --- | --- | --- |
| ***BRCA1*** | G360 cohort | cfDNA | 4.06 | [3.62-4.50] | 7707 | N/A |
| ***BRCA1*** | Chung et al [10] | metastatic tissue | 1.32 | [0.80-2.12] | 1816 | <0.001 |
| ***BRCA1*** | Mateo et al [8] | primary tissue | 1.71 | [0.00-3.64] | 175 | 0.118 |
| ***BRCA2*** | G360 cohort | cfDNA | 7.97 | [7.36-8.57] | 7707 | N/A |
| ***BRCA2*** | Chung et al [10] | metastatic tissue | 10.90 | [9.47-12.33] | 1816 | <0.001 |
| ***BRCA2*** | Mateo et al [8] | primary tissue | 8 | [3.98-12.02] | 175 | 0.987 |

G360: Guardant360 cohort, this study.

**Supplementary Figure 1.** Flow diagram of the cohort selection process. *VUS=Variant of Unknown Significance.


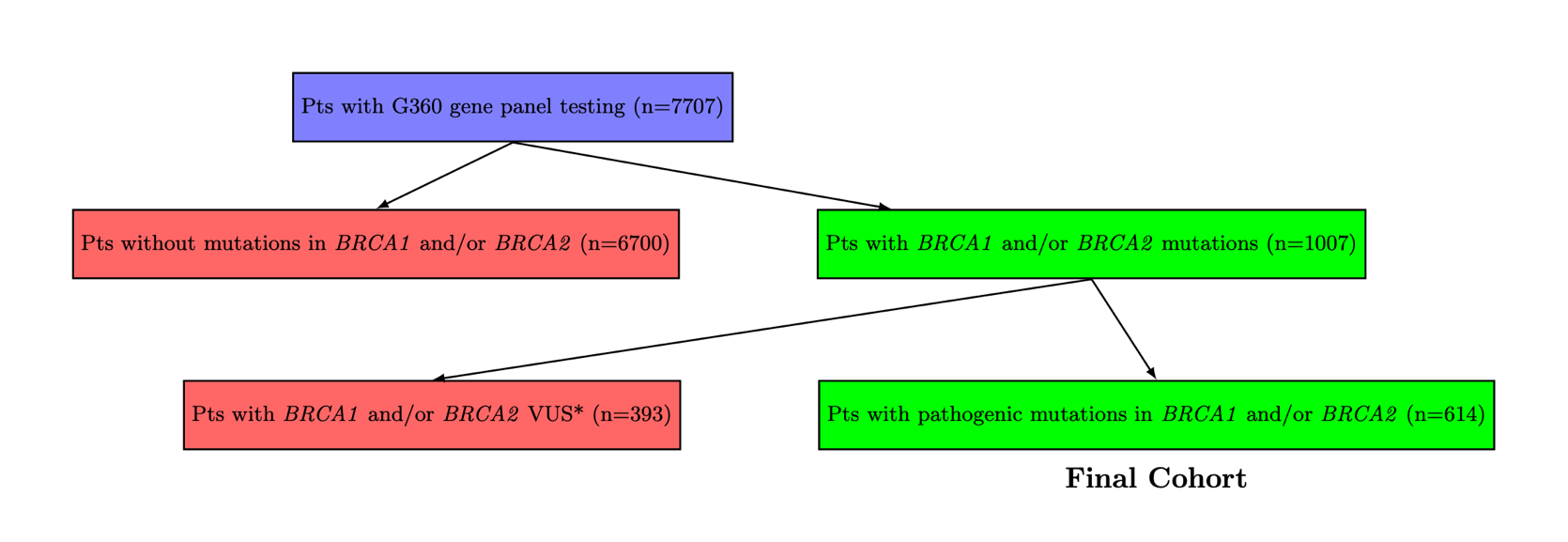

Supplement: Supplementary file 1 [file DataSheet_1.docx]
